# Construction of disulfidptosis-related lncRNA signature for predicting the prognosis and immune escape in colon adenocarcinoma

**DOI:** 10.1186/s12876-023-03020-x

**Published:** 2023-11-10

**Authors:** Pan Chen, Jun Yu, Qian Luo, Jie Li, Wei Wang

**Affiliations:** 1 Department of General Surgery, Nanjing Tongren Hospital, School of Medicine, Southeast University, Nanjing, 211102 China; 2 Department of Pediatrics, Affiliated Hospital of Nanjing University of Chinese Medicine, Taicang Hospital of Traditional Chinese Medicine, Taicang, 215400 China; 3 Department of Oncology, Second Affiliated Hospital, Nanjing Medical University, Nanjing, 210011 China; 4 Department of Clinical Laboratory, Lianshui County People’s Hospital, Huai’an, 223400 China

**Keywords:** Disulfidptosis, COAD, Immune Escape, Machine learning, Prognostic model

## Abstract

Colon adenocarcinoma (COAD) is one of the most frequent types of cancer worldwide. Disulfidptosis has been identified as a new mode of cell death recently. The goal of this study was to explore the possibility of a connection between disulfidptosis and COAD. RNA sequencing data from COAD patients were retrieved from the The Cancer Genome Atlas (TCGA) database for this investigation. R software and various methods were used to identify disulfidptosis-related lncRNAs (DRLs) in COAD, and a prognostic model was created based on 6 DRLs (AP003555.1, AL683813.1, SNHG7, ZEB1-AS1, AC074212.1, RPL37A-DT). The prognostic model demonstrated a good accuracy in predicting the prognosis of COAD patients, according to receiver operating characteristic (ROC) curve and Concordance index (C-index) analyses. Gene Ontology (GO) enrichment analysis and Kyoto Encyclopedia of Genes and Genomes (KEGG) enrichment analysis revealed significant differences in biological functions and signaling pathways involved in differential genes in risk subgroups, including protein − DNA complex subunit organization, Hippo signaling pathway, Wnt signaling pathway. TIDE analysis was done on risk groupings in this study, and it found that patients in the high-risk group had more immune escape potential and were less probable to react to immunotherapy. Real-time quantitative pcr (qRT-PCR) was used to identify the relatively high expression of 6 DRLs in colon cancer cell lines. In summary, 6 DRLs were identified as possible novel molecular therapy targets for COAD in this investigation. This prognostic model has the potential to be a novel tool for forecasting COAD prognosis in clinical practice, as well as providing new insights on the potential function and mechanism of disulfidptosis in the COAD process.

## Introduction

 COAD is one of the most common malignancies globally. Colon cancer has an annual average of 1,148,500 new cases and 576,800 new deaths, according to Global Cancer Statistics [1]. Because of the concealed signs of colon cancer, most patients are identified at an advanced stage. As a result, even after therapy, the prognosis for people with COAD is frequently poor [2]. In clinical practice, the prognosis of COAD patients is largely predicted by TNM staging, but the accuracy of prediction is not optimal [3]. It is critical to look for novel biomarkers to predict the prognosis of COAD patients.

By maintaining the secondary, tertiary, and quaternary structures of proteins, disulfides can improve their physical and chemical stability [4]. Liu et al. discovered a novel type of cell death known as disulfidptosis [5], which varies from other known types of death, such as necroptosis [6], pyroptosis [7], PANoptosis [8], NETosis [9]. When cells are starved, increased SLC7A11 expression causes disulfides to accumulate, resulting in disulfidptosis. The identification of a novel disulfidptosis mechanism provides new insights into the investigation of cell death.

Lncrnas have been demonstrated to have a crucial function in various kinds of malignancies. Yang et al. discovered that highly expressed LINC02159 in non-small cell lung cancer (NSCLC) tissue improved the stability of YAP1 mRNA via interacting with Aly/REF export factor (ALYREF) through m5C modification. Upregulated YAP1 can activate the Hippo and β-catenin signaling pathways, promoting NSCLC processes [10]. In artificially induced M2 human macrophages, Annika Karger et al. discovered that lncRNA ADPGK-AS1 was markedly elevated. Further research has revealed that ADPGK-AS1 is primarily found in mitochondria and binds to the mitochondrial ribosomal protein MRPL35, which promotes the tricarboxylic acid cycle and macrophage mitochondrial fission, resulting in macrophage M2 conversion and tumor growth [11]. Lncrna may be a significant prognostic indicator for COAD patients.

The relationship between lncrnas and disulfidptosis remains unclear, despite the fact that it has been demonstrated that both have a potentially significant function in COAD processes. In this work, 6 DRLs were found using integrated bioinformatics analysis and machine learning to develop a prognostic model for COAD patients. This model predicts the prognosis of COAD patients with good accuracy and is predicted to be an excellent prediction tool for forecasting the immunotherapy response and prognosis of COAD patients.

## Materials and methods

### Data acquisition

Four hundred seventy-six COAD tissue pairs and 41 normal tissue pairs, along with clinical data and RNA sequencing information, were acquired from the TCGA database (https://portal.gdc.cancer.gov/). Accessed 22 July 2023. Each tissue’s mRNA and lncRNA expression levels were extracted using Perl.

### Identification of disulfidptosis-related lncRNAs

The expression levels of disulfidptosis-related genes (GYS1, NDUFS1, OXSM, LRPPRC, NDUFA11, NUBPL, NCKAP1, RPN1, SLC3A2, SLC7A11) in each tissue were determined by employing R software. Least absolute shrinkage and selection operator (Lasso) regression analysis and the COX model were utilized to find 6 DRLs (AP003555.1, AL683813.1, SNHG7, ZEB1-AS1, AC074212.1, RPL37A-DT) in COAD tissues based on cor ≥ 0.4 and *p* value ≤ 0.001.

###  Construction of prognostic model

The riskscore of each COAD patient was determined using the formula risk = AP003555.1x0.3279 + AL683813.1x0.5689 + SNHG7x0.5197 + ZEB1-AS1x0.4394 + AC074212.1x0.5492 + RPL37A-DTx0.8707 (the value of lncRNAs is the level of expression of lncRNAs in each patient’s tissue). Patients were divided into high-risk and low-risk categories based on the median riskscore of all COAD patients.

###  GO enrichment analysis and KEGG enrichment analysis

Differentially expressed genes (DEGs) in risk subgroups were identified, based on logFC ≥ 1 and fdr < 0.05. Under the condition of *p* value < 0.05, GO enrichment analysis and KEGG enrichment analysis of DEGs were run using the R software package, including ComplexHeatmap, ggplot2, circlize, RcolorBrewer, ggpubr, clusterProfiler, org.Hs.eg.db, enrichplot, dplyr [12].

###  Cell culture

From the American Type Culture Collection (ATCC), human colon cancer cell lines (SW480, SW620, LOVO) and human normal intestinal epithelial cell (FHC) were purchased. All cells were cultivated using DMEM (Gibco, C11995500BT, USA), 1640 (Gibco, C11875500BT, USA) medium in a 5% CO2, 37 °C cell incubator (Thermo, USA). All mediums included 10% foetal bovine serum (FBS) (CLARK, FB25015, USA).

###  RNA extraction, RNA reverse transcription and qRT-PCR

TRIZOL (Life Technologies, Thermo Fisher, USA) was used to extract total RNA from cultivated cells. cDNA was produced by reverse transcription of total RNA using the kit (Vazyme, R323-01, China). Gene expression levels were measured by LightCycler (Roche, USA) and SYBR Green Master Mix (Vazyme, Q321-02, China). Based on 2^−ΔΔCT^, the relative expression was computed and standardized to GAPDH expression. Primer sequence: SNHG7: LEFT PRIMER: GCAAAGAGAAAGTGGCGATT, RIGHT PRIMER: AAGTGCCCGAGCTTCAGATA; ZEB1-AS1: LEFT PRIMER: AGAAGCATCGGCTGACAGAT, RIGHT PRIMER: GGTCCCAAAGACGTTTCCTTA; RPL37A-DT: LEFT PRIMER: GCCTCCTGAGAAATGTTTGC, RIGHT PRIMER: ATGGACCCAGAGATCAATGC; AC074212.1: LEFT PRIMER: CACCTTCGGATTCCAGGAGTT, RIGHT PRIMER: CCTCCACTTGGTACTAGCTGTAAGC; AL683813.1: LEFT PRIMER: GTGCTGTTCCCTAGCACGAT, RIGHT PRIMER: CATCGAGCAGACAAGTGAGG; AP003555.1: LEFT PRIMER: CAAGGGACCACACAGGAACT, RIGHT PRIMER: GGGACATCTGGAAGCCAGT; GAPDH: LEFT PRIMER: GAAGGTGAAGGTCGGAGTC, RIGHT PRIMER: GAAGATGGTGATGGGATTTC.

### Statistical analysis

All data analysis was done with R software and its tools. *P* < 0.05 was considered statistically significant.

## Results

### The identification of disulfidptosis-related lncRNAs

**Fig. 1:**
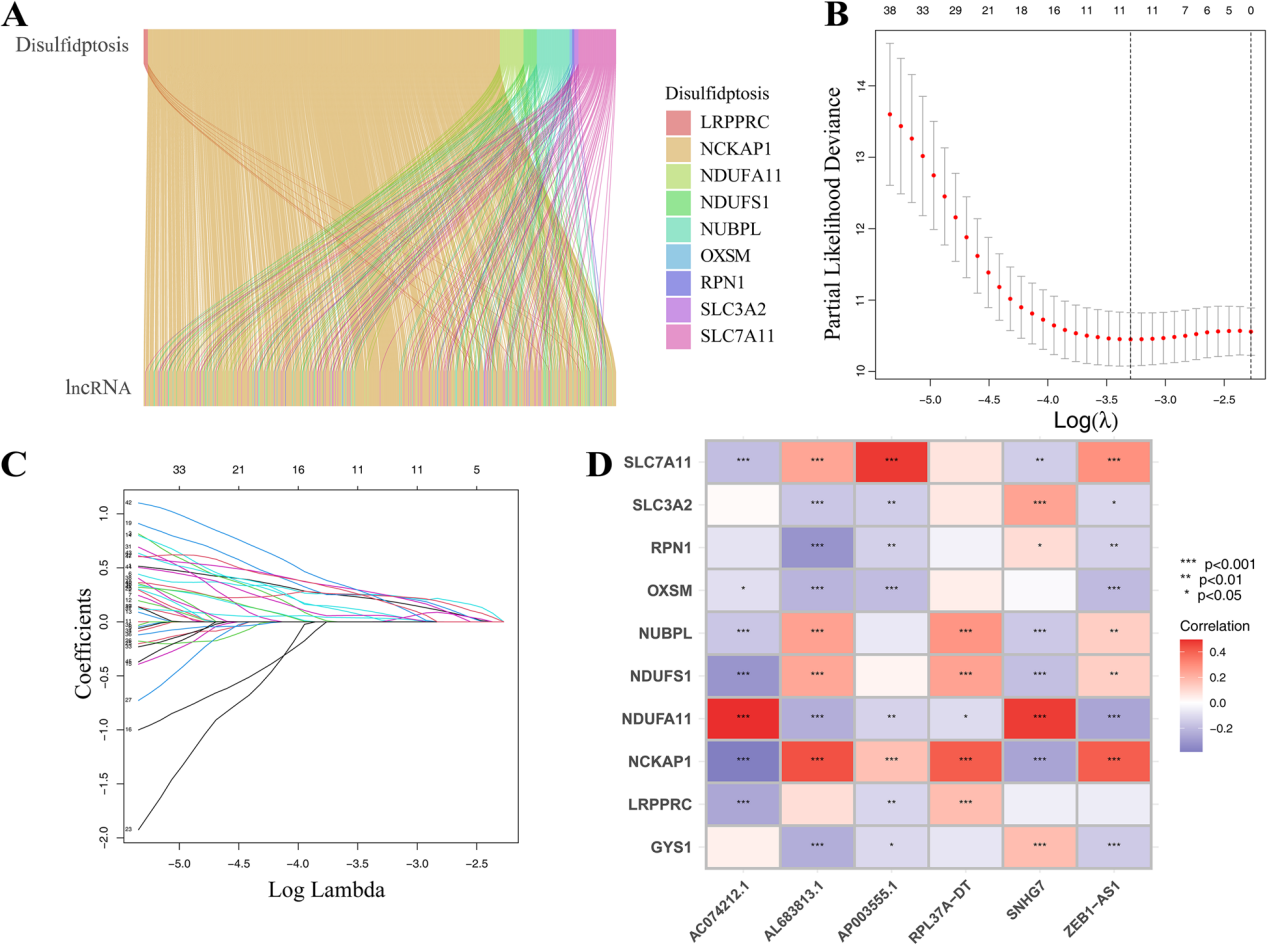
The identification of disulfidptosis-related lncRNAs. **A** Lncrnas associated with the disulfidptosis genes were identified in TCGA-COAD. Lasso regression analysis and the COX model **(B**-**C)** were used to identify 6 DRLs **D**

The expression levels of mRNAs and lncRNAs in each COAD patient were determined by downloading and re-annotating the RNA sequencing data of COAD patients (41 normal tissues and 476 COAD tissues) in the TCGA database. Further data analysis showed the expression levels of disulfidptosis-related genes (GYS1, NDUFS1, OXSM, LRPPRC, NDUFA11, NUBPL, NCKAP1, RPN1, SLC3A2, SLC7A11) in the TCGA-COAD dataset. According to the criteria of cor ≥ 0.4 and *p* value ≤ 0.001, disulfidptosis-related lncRNAs were screened (Fig. 1A). Lasso regression analysis and the COX model (Fig. 1B, C) were used to identify 6 DRLs for the construction of prognostic model (Fig. 1D; Table 1).

**Table 1:** Six disulfidptosis-related lncRNAs were utilized for constructing prognostic models

LncRNA	Coefficient	Hazard ratio	*P* value
AP003555.1	0.3279	1.480(1.140–1.921)	0.003
AL683813.1	0.5689	2.138(1.299–3.519)	0.003
SNHG7	0.5197	1.443(1.044–1.995)	0.026
ZEB1-AS1	0.4394	2.159(1.316–3.543)	0.002
AC074212.1	0.5492	1.804(1.017–3.199)	0.044
RPL37A-DT	0.8707	3.007(1.339–6.753)	0.008

### Construction prognostic model based on 6 disulfidptosis-related lncRNAs

**Fig. 2:**
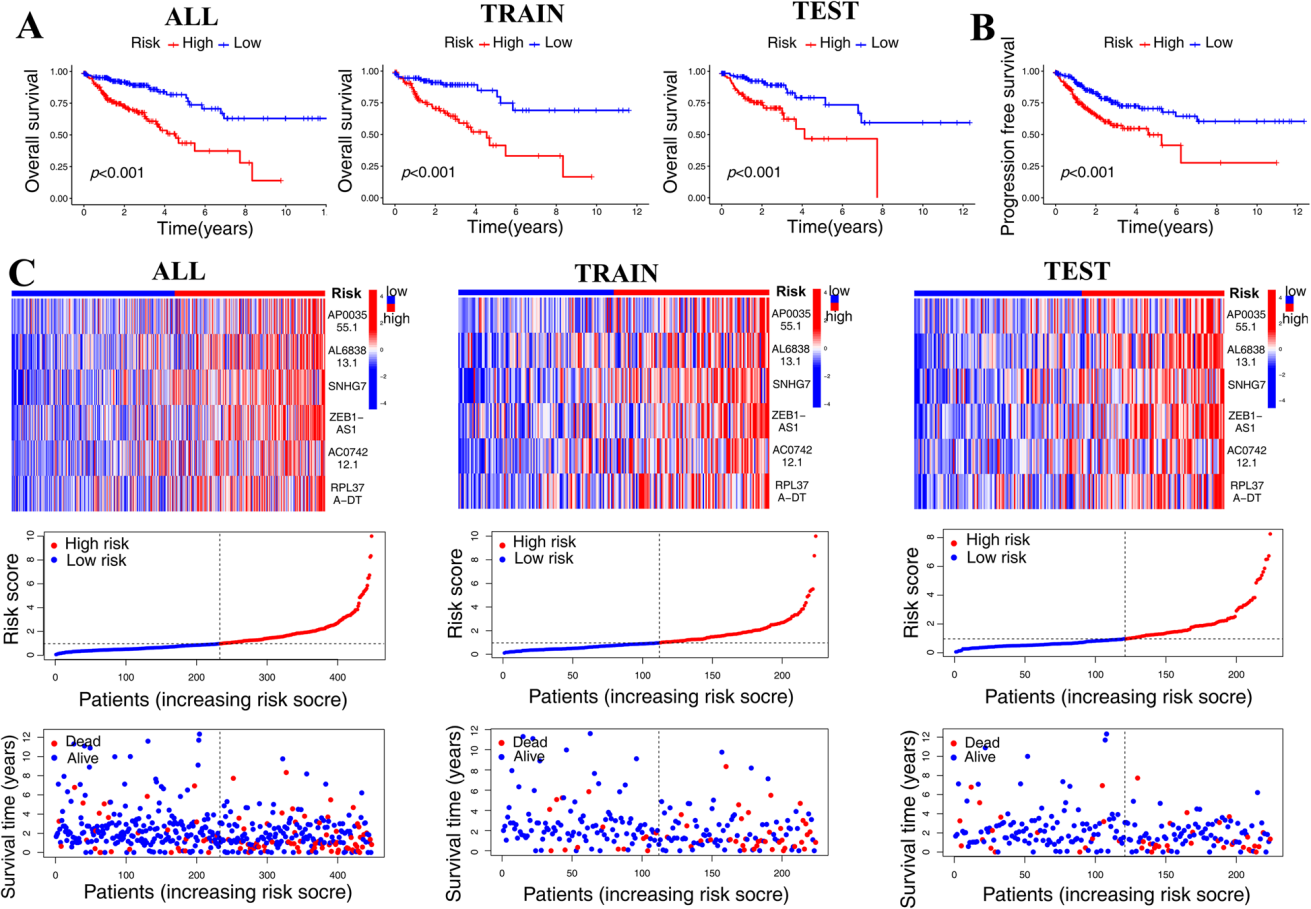
Construction and validation of prognostic model. **A** The KM survival analysis revealed substantial variations in OS between high-risk and low-risk individuals, whether in the all, train, or test groups. **B** Risk groupings’ PFS showed variation. **C** Patients in the high-risk group showed higher expression of 6 DRLs, and their survival times were shorter

Each patient’s risk score is determined using the formula of riskscore, and based on the median risk score of all patients, patients can be categorized into high risk or low risk groups. The TCGA-COAD dataset was used to acquire clinical information of patients. Patients were randomly assigned to the test group or the trian group, and there was no statistically significant clinical difference between the two groups (Table 2). The Kaplan-Meier (KM) survival analysis revealed substantial variations in overall survival (OS) between high-risk and low-risk individuals, whether in the all, train, or test groups (*p*<0.05) (Fig. 2A). Furthermore, there was a statistical difference between the high-risk and low-risk groups in terms of Progression Free Survival (PFS) (*p*<0.05) (Fig. 2B). Further investigation revealed that patients in the high-risk group had increased expression of 6 DRLs, and patient survival times were lower (Fig. 2C).

**Table 2:** Comparison of clinical characteristics between the train group and the test group

Covariates	Type	Total	Test	Train	*P* value
Age	≤ 65	183(40.94%)	84(37.5%)	99(44.39%)	0.1657
Age	>65	264(59.06%)	140(62.5%)	124(55.61%)	
Gender	FEMALE	214(47.87%)	105(46.88%)	109(48.88%)	0.7419
Gender	MALE	233(52.13%)	119(53.12%)	114(51.12%)	
Stage	Stage I	74(16.55%)	35(15.63%)	39(17.49%)	0.5962
Stage	Stage II	176(39.37%)	85(37.95%)	91(40.81%)	
Stage	Stage III	124(27.74%)	66(29.46%)	58(26.01%)	
Stage	Stage IV	62(13.87%)	35(15.63%)	27(12.11%)	
Stage	unknow	11(2.47%)	3(1.33%)	8(3.58%)	
T	T1	10(2.24%)	5(2.23%)	5(2.24%)	0.5923
T	T2	76(17%)	36(16.07%)	40(17.94%)	
T	T3	305(68.23%)	159(70.98%)	146(65.47%)	
T	T4	56(12.53%)	24(10.72%)	32(14.35%)	
M	M0	330(73.83%)	167(74.55%)	163(73.09%)	0.4799
M	M1	62(13.87%)	35(15.63%)	27(12.11%)	
M	unknow	55(12.3%)	22(9.82%)	33(14.8%)	
N	N0	265(59.28%)	127(56.7%)	138(61.88%)	0.4182
N	N1	102(22.82%)	52(23.21%)	50(22.42%)	
N	N2	80(17.9%)	45(20.09%)	35(15.7%)	

### Evaluating the prognostic model’s accuracy

**Fig. 3:**
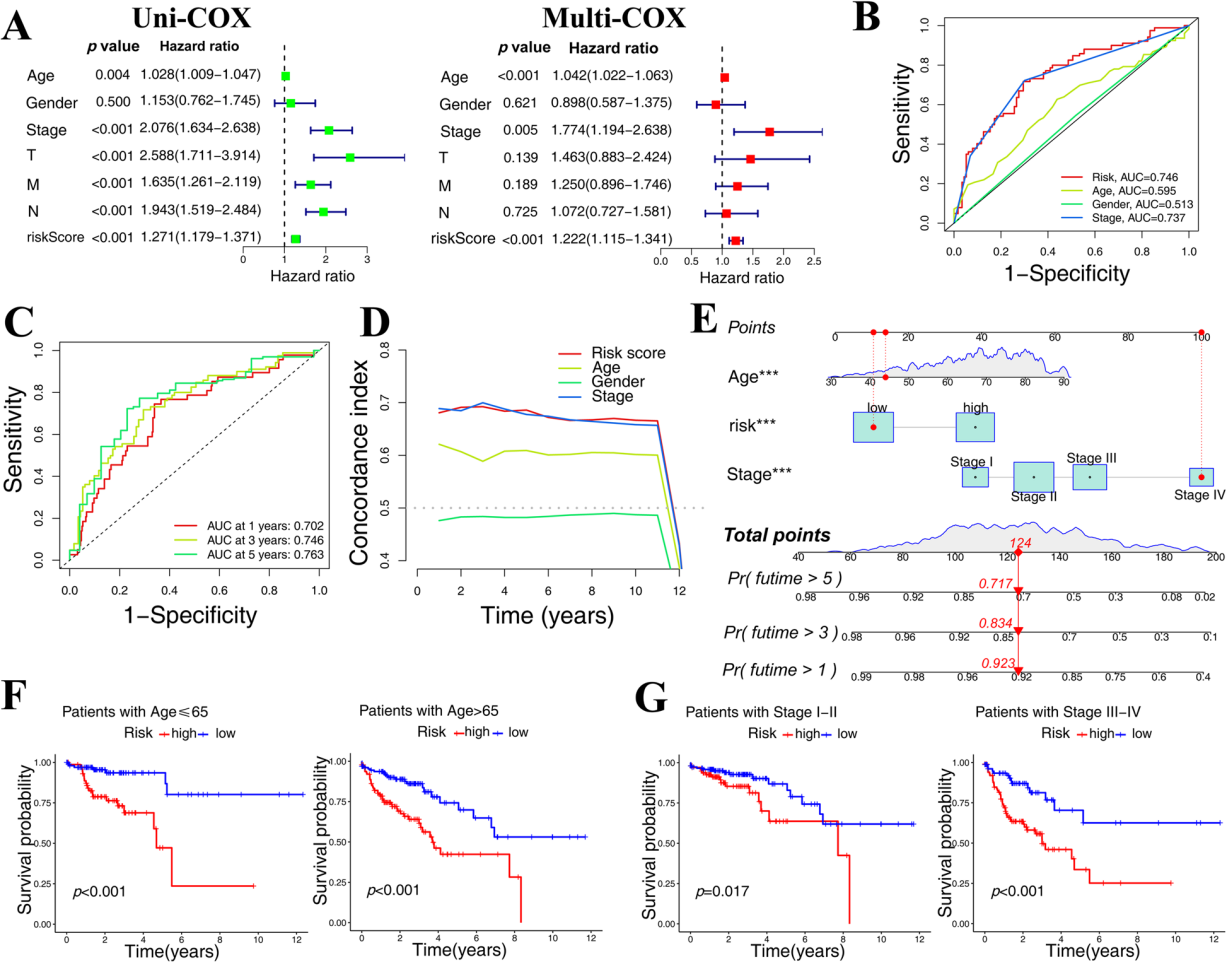
Evaluating the accuracy of the prognostic model. **A** Age, stage, and riskScore of TCGA-COAD patients were found to be independent prognostic markers in univariate and multivariate analyses. The prognostic model demonstrated higher sensitivity in predicting the prognosis of COAD patients, according to ROC curve (**B**, **C**) and C-index analyses (**D**). The 1-year, 3-year, and 5-year survival rates of COAD patients could be accurately predicted by nomogram **(E)**. The KM survival curves demonstrated that the prognostic model was appropriate for prognostic prediction in diverse clinical groups (**F**, **G**). Pr: probability

Univariate and multivariate analyses revealed that age, stage, and riskScore of TCGA-COAD patients might be employed as independent prognostic indicators (Fig. 3A). ROC curve (Fig. 3B, C) and C-index analyses (Fig. 3D) revealed that the prognostic model had greater sensitivity in predicting the prognosis of COAD patients than other clinical parameters (Age, Gender, Stage). Furthermore, based on the findings of univariate analysis and multivariate analysis, we created a Nomogram by assigning age, stage, and riskScore, and the results revealed that this Nomogram could reliably predict the 1-year, 3-year, and 5-year survival rates of COAD patients (Fig. 3E). The examination of the KM survival curves revealed that the prognostic model was suitable for prognostic prediction in various clinical groups (Fig. 3F, G).

###  GO enrichment analysis, KEGG enrichment analysis, immune function difference analysis and immune Escape prediction

**Fig. 4:**
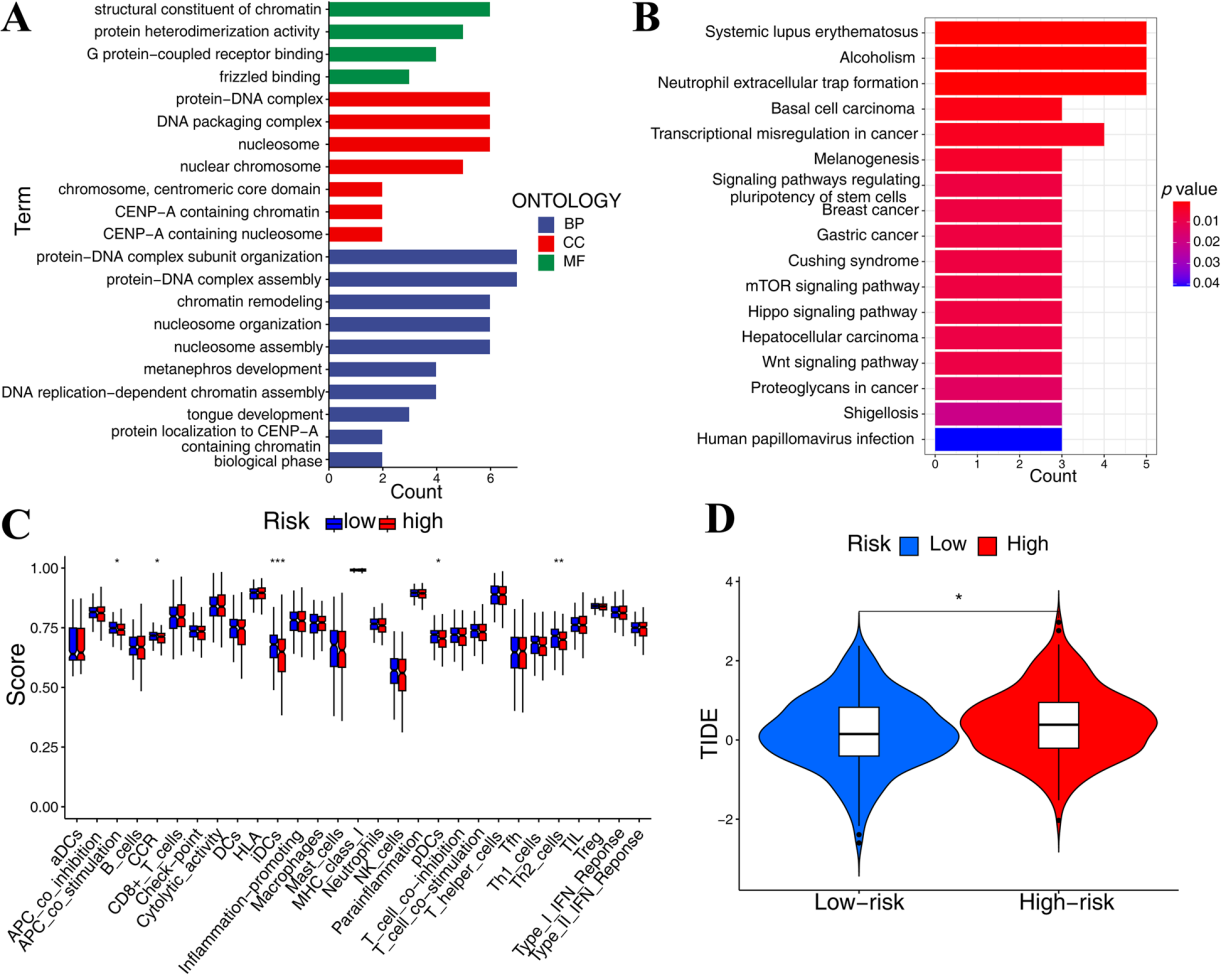
GO enrichment analysis, KEGG enrichment analysis, immune function difference analysis and TIDE analysis. GO (**A**) and KEGG (**B**) enrichment analyses revealed that DEGs in risk subgroups influenced several biological processes and cell signaling pathways of COAD. **C** Differential immune function analysis indicated differences between risk subgroups. **D** TIDE analysis found that the high-risk group was less sensitive to immunotherapy and had a higher incidence of immunological escape

The DEGs among risk subgroups were identified by employing differential expression analysis. According to GO enrichment analysis, the DEGs of risk categories were primarily involved in protein − DNA complex subunit organization, chromatin remodeling, structural constituent of chromatin (Fig. 4A). KEGG enrichment analysis revealed that differentially expressed genes in risk categories were mostly engaged in neutrophil extracellular trap formation, transcriptional misregulation in cancer, mammalian target of rapamycin (mTOR) signaling pathway, proteoglycans in cancer (Fig. 4B). Immune function differential study revealed variations in iDCs, Th2 cells, APC co-stimulation, CCR, and pDCs between the high-risk and low-risk groups (Fig. 4C). Furthermore, according to the TIDE prediction, the high-risk group was less responsive to immunotherapy and had a larger chance for immunological escape (Fig. 4D).

### Evaluating the expression levels of 6 disulfidptosis-related lncRNAs by qRT-PCR

**Fig. 5:**
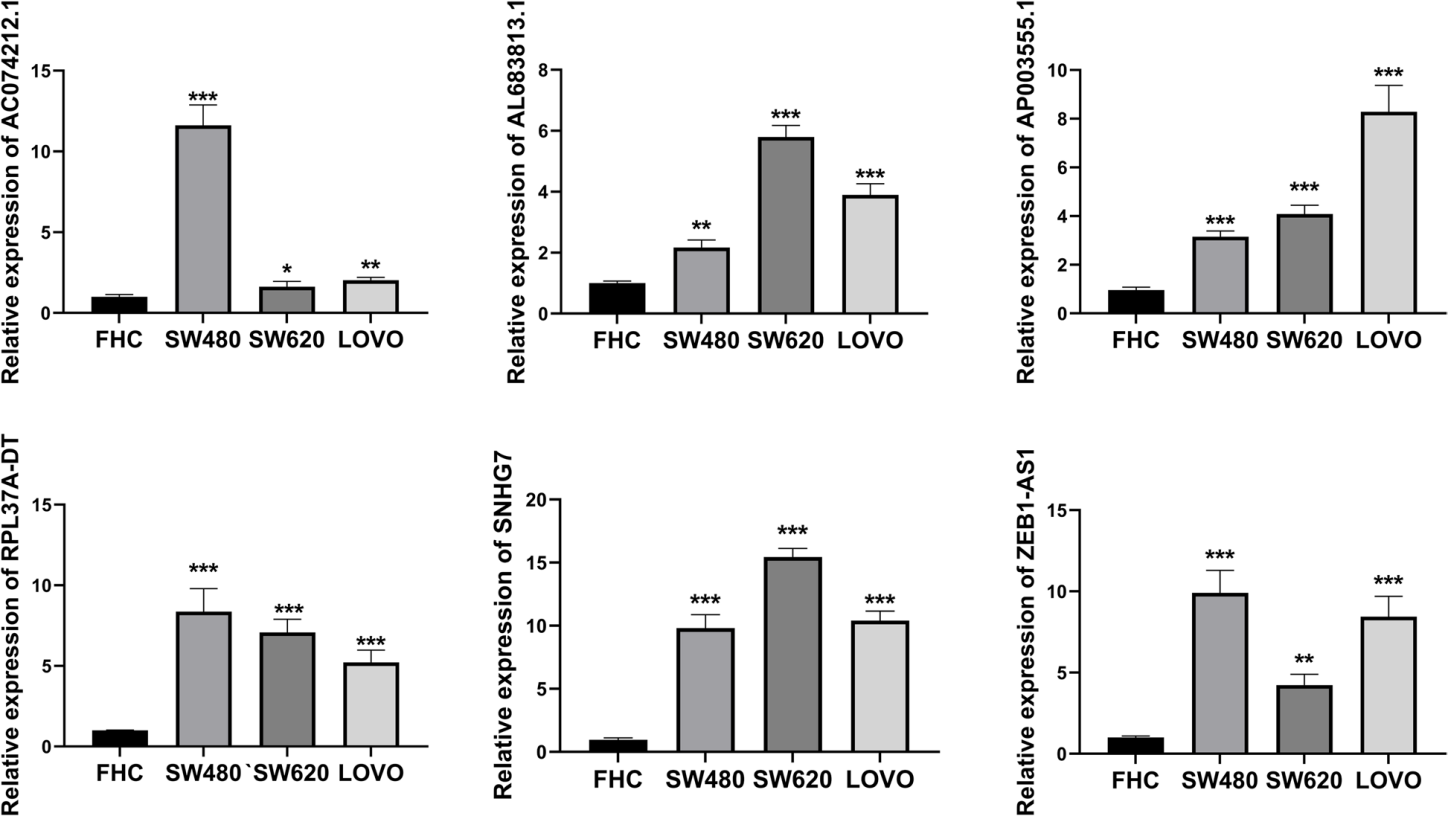
Identification of the expression level of DRLs in colon cancer cell lines

We utilized R software to identify 6 disulfidptosis-related lncRNAs that were particularly highly expressed in COAD patients for the construction of a prognostic model to predict the prognosis of COAD patients. We utilized qRT-PCR to confirm the findings and found that AP003555.1, AL683813.1, SNHG7, ZEB1-AS1, AC074212.1 and RPL37A-DT were considerably overexpressed in human colon cancer cell lines (SW480, SW620, LOVO) compared to normal intestinal epithelial cell (FHC) (Fig. 5).

## Discussion

The primary therapies for colon cancer include surgery, chemotherapy, and radiation therapy [13, 14]. Although the survival duration of certain colon cancer patients has increased due to the application of novel medicines such as molecular targeted therapy [15], immunotherapy [16, 17], and intestinal microbe management [18], many patients still have a poor prognosis. As a result, developing a novel prognostic model is beneficial in predicting the prognosis of COAD patients and subsequently developing individualized treatment for COAD patients. As a result, developing a novel prognostic model to reliably predict the prognosis of COAD patients is beneficial in developing individualized treatment for COAD patients.

Lasso regression analysis and the COX model were used in this work to identify 6 DRLs for the establishment of prognostic models, including AP003555.1, AL683813.1, SNHG7, ZEB1-AS1, AC074212.1, RPL37A-DT. Li et al. discovered that SNHG7, which is highly expressed in colorectal cancer, competes with GALNT1 for binding to miR-34a, resulting in increased GALNT7 mRNA expression. The PI3K/AKT/mTOR pathway is activated in colorectal cancer when GALNT7 is expressed at high levels, facilitating the disease spread [19]. ZEB1-AS1 is thought to play a significant role in colorectal cancer, according to several research [20, 21]. Lv et al. discovered that highly expressed ZEB1-AS1 in colorectal cancer may directly target and limit the production of miR-181a-5p, which results in increased levels of β-catenin and the transcription factor TCF4 which facilitate the development of CRC [22]. AP003555.1 has been known to be associated with ferroptosis and oxidative stress in colorectal cancer [23, 24], however there has been no investigation into the link with disulfidptosis. AC074212.1 has been identified to be involved in tumor immunity and to contribute to the prognostic prediction of endometrial carcinoma [25], but no research in colorectal cancer have been undertaken. Furthermore, the possible role and mechanism of RPL37A-DT and AL683813.1 in cancers are unknown and need to be investigated further. 6 DRLs have the potential to be novel therapeutic targets and biomarkers for colorectal cancer.

Many investigations have demonstrated that the formation and progression of malignancies is frequently associated by immune escape [26–29]. Immunotherapy is also gaining popularity in cancer treatment [30–33]. Immunotherapy can considerably enhance the overall survival of many cancer patients, but failure to respond effectively to immunotherapy in some individuals frequently leads in poor treatment outcomes [34–39]. Predicting a patient’s reaction to immunotherapy might aid in the development of more accurate therapies for patients. TIDE analysis was done on risk groupings in this study, and it found that patients in the high-risk group had more immune escape potential and were less probable to react to immunotherapy. These findings imply that this predictive model might offer some recommendations on whether COAD patients should undergo immunotherapy.

## Conclusion

In general, 6 DRLs were selected for the establishment of COAD prognostic models in this study. This model shows great accuracy in predicting the prognosis of COAD patients and has the ability to provide guidance for COAD therapy in clinical practice.

## Data Availability

The datasets analysed during the current study are available in the TCGA databases (https://portal.gdc.cancer.gov/). Accessed 22 July 2023. In the “Materials and methods” section, the analysis methods and software applications are described. All other R code and analyses are available from the corresponding author upon request.
